# Unsupervised machine learning to investigate trajectory patterns of COVID-19 symptoms and physical activity measured via the MyHeart Counts App and smart devices

**DOI:** 10.1038/s41746-023-00974-w

**Published:** 2023-12-22

**Authors:** Varsha Gupta, Sokratis Kariotis, Mohammed D. Rajab, Niamh Errington, Elham Alhathli, Emmanuel Jammeh, Martin Brook, Naomi Meardon, Paul Collini, Joby Cole, Jim M. Wild, Steven Hershman, Ali Javed, A. A. Roger Thompson, Thushan de Silva, Euan A. Ashley, Dennis Wang, Allan Lawrie

**Affiliations:** 1 Singapore Institute for Clinical Sciences, Agency for Science Technology and Research (A*STAR), Singapore, Republic of Singapore; 2 Bioinformatics Institute, Agency for Science Technology and Research (A*STAR), Singapore, Republic of Singapore; 3 Department of Neuroscience, University of Sheffield, Sheffield, UK; 4 Department of Infection, Immunity and Cardiovascular Disease, University of Sheffield, Sheffield, UK; 5 Department of Computer Science, University of Sheffield, Sheffield, UK; 6 National Heart and Lung Institute, Imperial College London, London, UK; 7 Department of Nursing, Faculty of Applied Medical Sciences, Taif University, Taif, Saudi Arabia; 8 Insigneo Institute for in-silico Medicine, University of Sheffield, Sheffield, UK; 9 Department of Cardiovascular Medicine, Stanford University, Stanford, CA USA

**Keywords:** Biomarkers, Predictive markers

## Abstract

Previous studies have associated COVID-19 symptoms severity with levels of physical activity. We therefore investigated longitudinal trajectories of COVID-19 symptoms in a cohort of healthcare workers (HCWs) with non-hospitalised COVID-19 and their real-world physical activity. 121 HCWs with a history of COVID-19 infection who had symptoms monitored through at least two research clinic visits, and via smartphone were examined. HCWs with a compatible smartphone were provided with an Apple Watch Series 4 and were asked to install the MyHeart Counts Study App to collect COVID-19 symptom data and multiple physical activity parameters. Unsupervised classification analysis of symptoms identified two trajectory patterns of long and short symptom duration. The prevalence for longitudinal persistence of any COVID-19 symptom was 36% with fatigue and loss of smell being the two most prevalent individual symptom trajectories (24.8% and 21.5%, respectively). 8 physical activity features obtained via the MyHeart Counts App identified two groups of trajectories for high and low activity. Of these 8 parameters only ‘distance moved walking or running’ was associated with COVID-19 symptom trajectories. We report a high prevalence of long-term symptoms of COVID-19 in a non-hospitalised cohort of HCWs, a method to identify physical activity trends, and investigate their association. These data highlight the importance of tracking symptoms from onset to recovery even in non-hospitalised COVID-19 individuals. The increasing ease in collecting real-world physical activity data non-invasively from wearable devices provides opportunity to investigate the association of physical activity to symptoms of COVID-19 and other cardio-respiratory diseases.

## Introduction

Exposure to SARS-CoV-2 can result in a broad spectrum of symptoms ranging from asymptomatic infection to severe COVID-19 requiring hospitalisation. In the most severe cases this can lead to death (20–30%)^1^. Some patients recovering from COVID-19 of all severities are now well recognised to have long-lasting effects, including fatigue, dyspnea, and neuropsychological symptoms^1–3^. This post-COVID-19 syndrome termed Long-COVID-19 is defined by the UK National Health Service (NHS) as continuous unexplained symptoms for more than 12 weeks after the onset of symptoms (National Institute for Health and Care Excellence, 2020). Data from the UK COVID-19 Symptom Study (Zoe) app has reported an ongoing self-reported symptom burden in over 4000 incident cases^4^. The UK’s Office for National Statistics (ONS) confirmed long-term COVID-19 symptoms for more than 12 weeks in 13.7% of the 20,000 people who tested positive between April 2020 and March 2021^5^, however, the reported prevalence Long-COVID-19 varies from 5–50% of those reporting symptoms^6^.

A wide spectrum of persistent post-acute sequelae of SARS-CoV-2 infection (PASC)-associated symptoms have been recognised to persist in patients hospitalised with severe COVID-19 following infection. Most frequent individual symptoms are fatigue and dyspnoea (at 1–4 months), with many patients (75%) experiencing at least one symptom at up to 12 months^7^.

During the COVID-19 pandemic frontline healthcare workers (HCW) were at a high risk of infection. This resulted in several local and national studies to monitor infection and symptoms. Studies from UK NHS trusts reported SARS-CoV-2 seroprevalence rates of up to 50% in frontline HCW after the first-wave of the COVID-19 pandemic, at a time when the estimated seropositivity in the general population was only 6%^8,9^. A longitudinal study of the long-term symptoms of COVID-19 in 38 HCW indicated that 55% indicated at least one continuous symptom, with fatigue being the most abundant symptom (57%) six months after COVID-19 diagnosis^10^. Within the population there is a large degree of heterogeneity in COVID-19 both in terms of severity and duration of symptoms^4^. The long-term outcomes of those with asymptomatic or mild COVID-19 is less well-defined.

There has been heightened interest in the use of smartphones and wearable devices to capture real-world longitudinal data during the pandemic^11^. The utility of smartphones and wearable devices to monitor physiological signs as predictor of COVID-19 has previously been reported^12^, and the combination of symptoms and activity data has been shown to distinguish COVID-19 positive participants better than symptoms alone^13^, and even before the onset of COVID-19^14^. Previous studies have highlighted the potential for heart rate variability (HRV) to predict survival in hospitalised patients with COVID-19^15^. Even in the absence of self-reported symptoms the use of predictive algorithms that utilise smartwatch data has been demonstrated as a good predictor of COVID-19^16^, and for the ability to distinguish between influenza and COVID-19^17^. Most studies have focussed on identifying the diagnosis of COVID-19 using physical activity parameters as surrogate variables (measured via a smart watch). However, there is growing interest, and evidence for the utility of smartwatches and other wearable technology to detect COVID-19 as well other cardio-respiratory infections/diseases is in the early stages^18^. However, little is known about the utility of HRV, or other measurements of cardiovascular or physical activity in relation to mildly symptomatic people infected with SARS-CoV-2^11,19^. In this study, we propose that unsupervised modelling of trajectories could identify patterns in COVID-19 symptoms that were associated with long- and short-COVID-19 in this non-hospitalised cohort. Our second aim was to assess whether there were distinct trajectory patterns of physical activity, and thirdly, whether there was any association between COVID-19 and physical activity trajectory patterns.

We present data from a cohort of HCWs with at least one-year follow-up after confirmed SARS-CoV-2 infection whose symptoms and physical activity levels were recorded at clinic visits and via the MyHeart Counts Study iOS App^20^. The different data structure, and frequency of collection required a different technical approach to identify trends in each dataset. The aim was to devise a methodology to identify trends in the resolution of symptoms over time following the diagnosis of COVID-19 and test whether this was associated with physical activity. Although the sample size was small, we found a weak association between COVID-19 symptom trajectories and markers of real-world of physical activity, specifically the distance the user has moved by walking or running (distanceWalkingRunning).

## Results

### Hospital workers with COVID-19

**Fig. 1:**
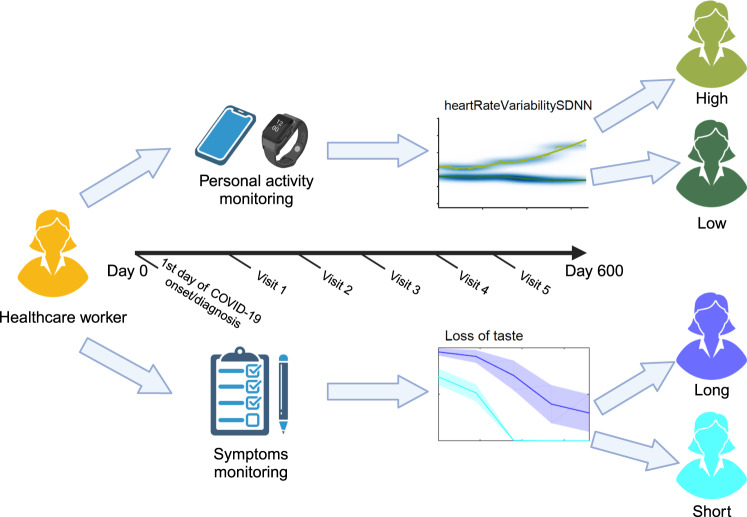
Workflow of study to track COVID-19 symptoms and physical activity of healthcare workers over a 600-day period. Physical activity data was collected using HealthKit on iOS devices (iPhones and Apple Watches) from the first day of COVID-19 symptom onset, serology test or PCR diagnosis. Current and previous symptoms were self-reported at clinical visits. Unsupervised machine learning methods were applied to the longitudinal activity (e.g. heart rate variability (heartRateVariabilitySDNN)) and symptoms (loss of taste) to classify each individual healthcare worker as having high/low physical activity (light green/dark green) and long/short COVID-19 symptoms (dark blue/light blue). We can subsequently taste whether these activity and symptom patterns are associated.

204 HCW participants were recruited to the study between July 2020 and July 2021 (Table 1). Of those recruited, 121 participants had either been PCR-positive or were seropositive for SARS-CoV-2 spike and nucleocapsid at the time of consent and had a history of COVID-19 symptoms. The remaining 17 had a confirmed PCR-positive or seropositive test, but with no reported symptoms, 65 participants were negative for COVID-19 and 1 participant did not have information about PCR or seropositive test. Participant demographics and data on COVID-19 testing, symptoms, vaccination were recorded during clinician led clinics, and 140 participants who owned compatible iPhone and were recruited into the MyHeart Counts App Study (Fig. 1). Of those, 3 participants already owned an existing compatible smartwatch, and 51 participants were provided with an Apple Watch Series 4 as part of the study. The proportion of individuals who had a comorbidity was higher in infected individuals and those with activity data (50.4% and 61.9%) compared to the full cohort (42.6%). A list of underlying medical conditions affecting more than two participants is shown in Supplementary Table 1.

**Table 1:** Demographics and summary statistics of symptoms and activity data.

Statistics measures	Values	Full cohort of HCW		Had COVID-19 symptoms		Had activity data from wearables		Had COVID-19 and activity data	
		*n*	Value	*n*	Value	*n*	Value	*n*	Value
Age (Yrs)	Mean ± SD	204	44.5 ± 10.85	121	45.51 ± 10.25	34	42.8 ± 10.30	21	43.5 ± 11.13
Gender (%)	Female	125	61.3	106	87.6	25	73.5	16	76.2
	Male	29	38.7	15	12.4	9	26.5	5	23.8
Ethnicity (%)	Other	23	11.3	18	14.9	4	11.8	3	14.3
	White British	181	88.7	103	85.1	30	88.2	18	85.7
Recorded comorbidity (%)	No	117	57.4	60	49.6	19	55.9	8	38.1
	Yes	87	42.6	61	50.4	15	44.1	13	61.9

### COVID-19 symptoms and identification of long- and short-COVID-19 trajectories

**Fig. 2:**
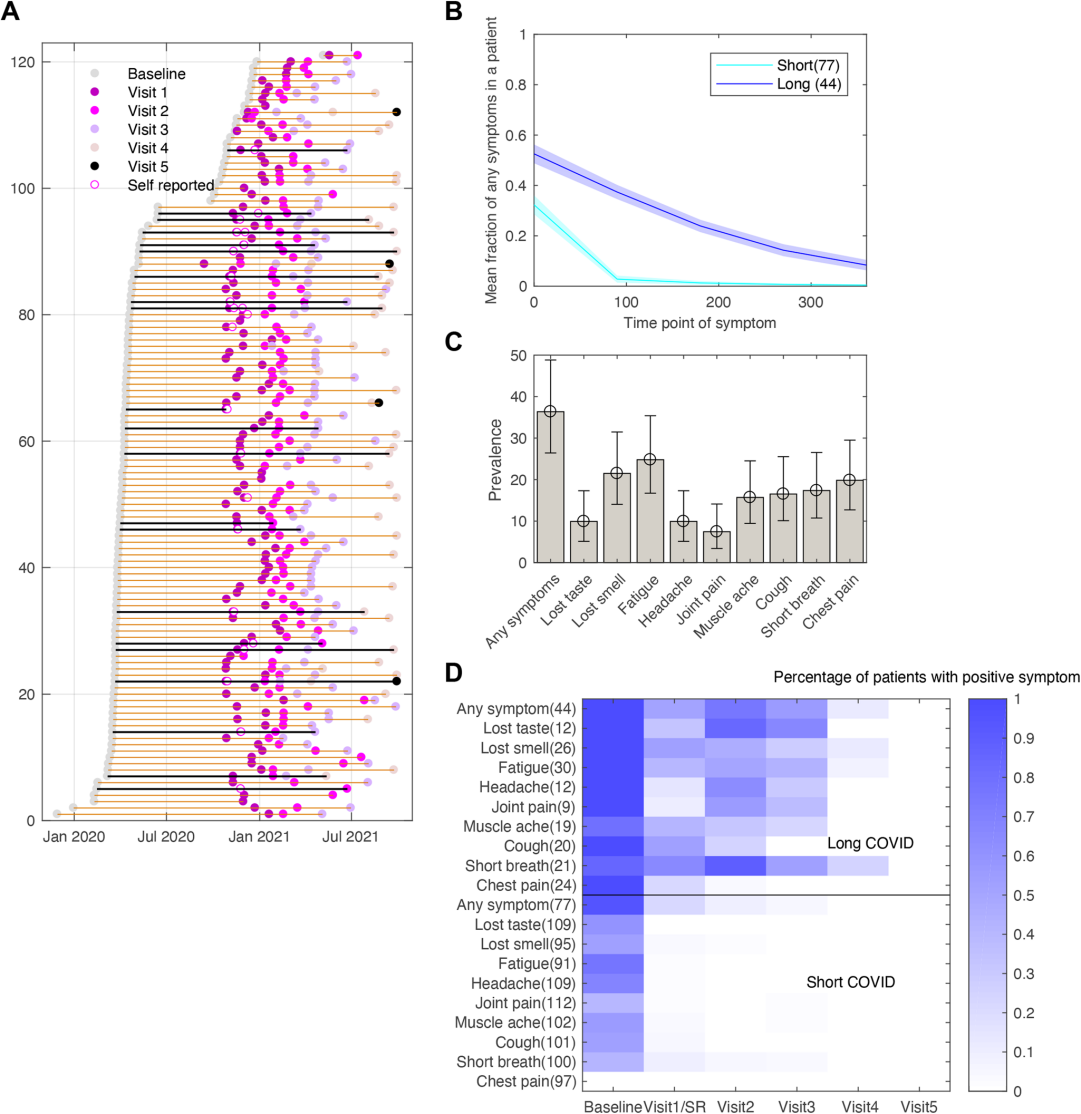
Classification of reported COVID-19 symptom trajectories of 121 HCWs. **A** Timeline of visits for each participant when the symptoms were reported. Black lines represent the patients who had physical activity data recorded as well. **B** Average estimated probability (with 95% confidence interval) of presence of any symptom over time of participants who were grouped as having short or long trajectory patterns. **C** Prevalence of the long-term symptom patterns along with 95% confidence interval. **D** Heatmap showing the probability of presence of a symptom at a particular visit for individuals in either the long or short COVID-19 trajectory groups. Note that there were no patients with positive symptoms at visit 5 and self-reported symptoms were combined with visit 1 symptoms data as they had overlapping dates. Number of patients shown in parentheses.

COVID-19 symptoms were recorded over five clinic visits spread over more than one year from 29 July 2020 (first clinic visit) to 29 September 2021 (last clinic visit) for different participants (Fig. 2A). The median time between reported COVID-19 positive test and the first follow-up visit was 227 [4–418] days and time to the last follow-up visit was 408 [41–564] days. In addition to the clinical visits, participants also uploaded the self-reported symptoms via the MyHeart Counts App at different timepoints within the period of monitoring of COVID-19 symptoms (Fig. 2A). In total, 17 symptoms were reported at clinic visits comprising fever, lost taste, lost smell, fatigue, confusion, nausea or vomit, headache, wheeze, sore throat, abdominal pain, joint pain, runny nose, muscle ache, cough, short breath, diarrhoea, and chest pain. The number of self-reported symptoms was resultant upon answering 11 questions related to the presence or absence of fever, lost smell, fatigue, nausea or vomiting, sore throat, muscle ache, cough, short breath, diarrhoea, chest pain, loss of appetite and were asked in the context of previous week. Amongst the 11 self-reported symptoms, only 10 symptoms formed a subset of the clinically reported symptoms and were used in estimation of symptoms trajectories. The ‘loss of appetite symptom’ did not coincide with the questions in clinically reported symptoms and therefore was dropped from trajectory estimation. There were 138 participants who reported symptoms at a minimum of two timepoints (clinical or self-reported) including the baseline symptoms at the time of diagnosis. Of these 138 participants, 116 had a date of onset of symptoms and five had a date of positive COVID-19 PCR that was used as to define the onset of COVID-19 resulting in 121 participants for analysis of symptom trajectories (Fig. 2A).

Using an unsupervised clustering method, latent class growth analysis, we tested two and three class solutions and found two-class solution to have the highest posterior probability across symptoms tested (Supplementary Table 2) with 77 and 44 participants in each trajectory class. When we look at the presence of any symptom, 44 participants had a significantly higher proportion of any symptom for a longer period compared to the second group of 77 participants who had a symptomatic trajectory for a shorter period (Fig. 2B).

As a sensitivity analysis, we excluded patients whose baseline onset of symptoms was after 1 July 2020 and had only short COVID-19 patterns as observed in our classification. We performed the classification corresponding to total symptoms and found that there were 97 individual trajectories remaining which resulted in two groups of 53 short and 44 long trajectories. Compared to the previous analysis with 121 subjects, only one trajectory was classified in a different group, reflecting the robust classification of long trajectories which were not influenced by the changes in the number of short trajectories (Supplementary Fig. 1). We performed down-sampling of symptoms data to test the robustness of the trajectory classification. We found that separate patterns of trajectories could be identified for even using as little as 40% of the total participants (Supplementary Fig. 2). We extrapolated the probability of observing symptoms at equal time intervals spread over a year, which allowed us to approximately match the number of visits and the follow-up duration of symptoms. When we changed the duration and number of time intervals from which the symptom probabilities were calculated, there was still agreement with the original results (Cohen’s Kappa ≥0.97; Supplementary Fig. 3; Supplementary Table 3), suggesting that the clustering patterns were robust and reproducible.

For each specific symptom, we also tested a two-class solution to group individual trajectories. Evidence of two classes (posterior probability >0.95) was observed in all nine symptoms (Supplementary Fig. 4 and Supplementary Table 2) with an uneven distribution of trajectories between the two classes: ‘lost taste’ (109/12), ‘lost smell’ (95/26), ‘fatigue’ (91/30), ‘headache’ (109/12), ‘joint pain’ (112/9), ‘muscle ache’ (102/19), ‘cough’ (101/20), ‘short breath’ (100/21) and ‘chest pain’ (97/24). The other eight symptoms measured, including fever, nausea, wheeze, confusion, sore throat, abdominal pain, runny nose, and diarrhoea did not show any evidence of existence of classes. Many of these symptoms had reported only at baseline, or a very small fraction of patients reported those symptoms at subsequent timepoints. The prevalence of long COVID-19 trajectories of any reported symptom is 36.36% [26.42,48.82] with fatigue being the most prevalent reported symptom with frequency of 24.79% [16.73, 35.39] (Fig. 2C) which is in agreement to the meta-analysis of severe-COVID-19 infection symptoms and the description of PASC^7^. In Supplementary Table 4, we observed that the prevalence of long COVID-19 patterns identified using an unsupervised clustering method in our study is consistent with other reported studies. The heatmap presented in Fig. 2D shows that in long COVID-19 trajectory patterns, the fraction of patients with any reported positive symptoms or a specific symptom is higher than in the short trajectory patterns at each visit. It is to be noted that at visit 5, there were only four patients and none of them reported any positive symptoms.

### Association of symptoms trajectories with demographics and health factors

**Fig. 3:**
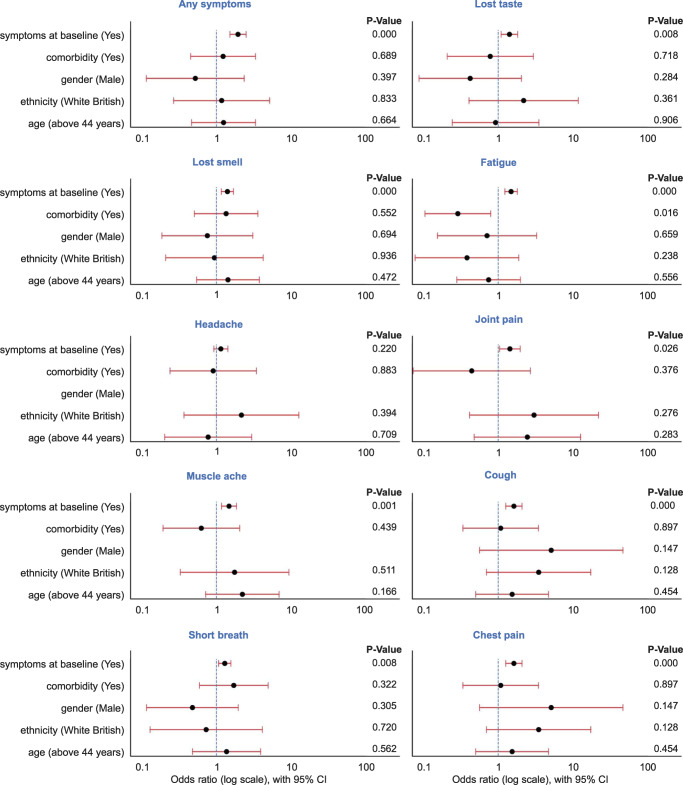
Odds ratios of long vs short COVID-19 symptoms. Forest plot showing odds of different parameters associated with long trajectory patterns of any or specific reported symptoms. Gender variable that shows no bar and *p*-value in headache, joint pain, and muscle ache symptoms because there were not enough male individuals to calculate odds. *n* = 121 participants.

Next, we examined the demographical differences between the two groups of trajectories. There was a similar gender imbalance in both groups with 69 female and 8 male participants who had short COVID-19 trajectory patterns, compared to 37 females and 7 males with long COVID-19 trajectory patterns. The mean age in short and long trajectory patterns in any positive symptoms was (45.2, 46.0) ± standard deviation (10.0, 10.7), respectively. Amongst the participants with short trajectory patterns, there were 66 White British and 11 other ethnicities while in the long trajectory pattern group there were 37 White British and 7 other ethnicities. Ethnicity did not play a significant factor in the presence of symptoms between the two categories of trajectories, but we may be underpowered given that <15% of participants were non-White British. We did not see any significant associations of comorbidity, gender, ethnicity, and age with long and short classes corresponding to any symptoms or the individual symptom classes. The most significant finding was that individuals with a higher number of symptoms at baseline had higher odds of having long symptom trajectories (Fig. 3, Supplementary Table 5).

### Association of symptoms trajectories with occurrence of re-infection

COVID-19 reinfections were observed in 38 participants from July 2021 till June 2022 and (24 short, 14 long trajectories of any symptoms). Amongst the 38 participants, four reported a second re-infection. For these four patients, we considered the data corresponding to the nearest visit from the date of the first re-infection. Amongst all 38 reinfected patients, we calculated the odds ratio of recurrence of 17 specific reported symptoms for individuals with long trajectories as compared to short trajectories. The symptoms that recurred which were associated with higher mean odds of being in long trajectory patterns of any symptoms, were nausea or vomit, joint pain, and muscle ache 21.0 [1.035, 426.0], 12.8 [1.31–125.1] and 6.071 [1.412–26.03], respectively; Supplementary Table 6).

### Physical activity trajectories during and after COVID-19 symptoms

**Fig. 4:**
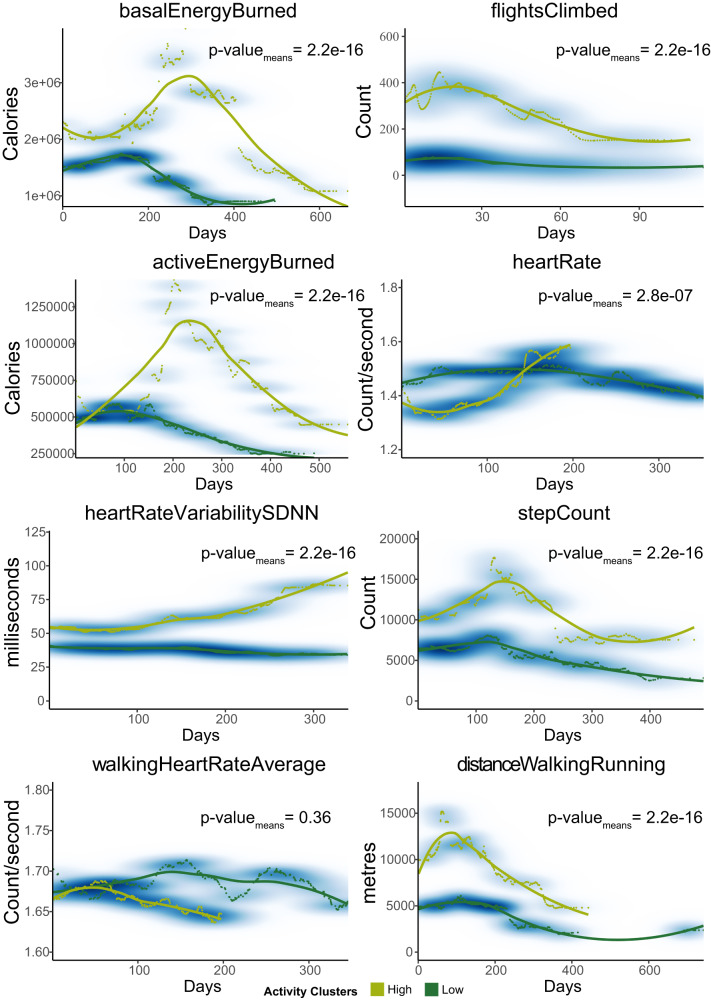
Physical activity trajectories of 34 HCWs following COVID-19 onset. Graphs show the smoothed mean representative trajectories of each generated cluster for the physical activity measures. The mean representative trajectories of each cluster show the separation of high and low activity trajectories. High activity is coloured light green and low activity is dark green. The individual timepoints of the cluster members are visible around the curves along with a density estimation in blue. Activity definitions are from Apple Healthkit: basalEnergyBurned (resting energy burned by the individual), flightsClimbed (number flights of stairs that the individual has climbed), activeEnergyBurned (active energy burned by the individual), heartRate (average heart rate), heartRateVariabilitySDNN (standard deviation of heartbeat intervals of the individual), stepCount (number of steps the individual has taken), walkingHeartRateAverage (individuals average heart rate while walking), distanceWalkingRunning (distance the individual has moved by walking or running). *p*-values are reported for each activity using a Welch Two Sample t-test, *n* for each activity for high and low activity clusters is provided in Supplementary Table 10.

Two clusters of participants were formed for each physical activity with cluster sizes and the statistics for the time-points used (overall average 487 time-points) presented in Supplementary Table 7. For each activity measured, the 34 participants with wearables were split between two clusters resulting in the low activity clusters being on average three times larger in each case. Clustering of distanceWalkingRunning (distance moved by walking or running) trajectories generated the most similar sized clusters of 18 with low activity and 13 with high activity, whereas the number of flightsClimbed (number flights of stairs climbed) had 28 with low activity and 4 with high activity. There were significant differences in the average trajectory corresponding to each activity (*p*-values <= 2.858e−07), except for the walkingHeartRateAverage (average heart rate while walking) (*p*-value = 0.3593) (Fig. 4; Supplementary Table 8). More prominently, patients in the high activity cluster showed approximately double the distanceWalkingRunning measure (10,955 m) as well as activeEnergyBurned (active energy burned by the individual) (949,650 calories) compared to the low cluster members (4964.5 m and 538,190 calories, respectively, with *p*-values of 2.2e−16). The highest difference was the number of flightsClimbed where high activity participants climbed 243 more flights, especially during the first 30 days (Fig. 4). While we could identify different trajectory clusters for most activities, differences for heartRate (individual’s average heart rate) were not significant.

As shown in Supplementary Table 7, all activity measures have a similar total amount of timepoints, but their spread across time is different as asynchronous gaps were created by the difference in when data from the wearable devices were recorded. However, this did not influence the clustering algorithm as the Fretchet-based formula that we used considers the overall shape of the longitudinal curves rather than individual timepoint-to-timepoint comparisons across the patient clusters. Following the same reasoning, the missing data values did not influence the clustering process. Finally, we down-sampled the number of participants with activity data to observe the effects of longitudinal clustering with progressively smaller datasets. We were still able to observe distinct high and low trajectory patterns for flightsClimbed as we reduced the number of samples used (Supplementary Fig. 5).

### Association of symptoms and physical activity trajectories

**Fig. 5:**
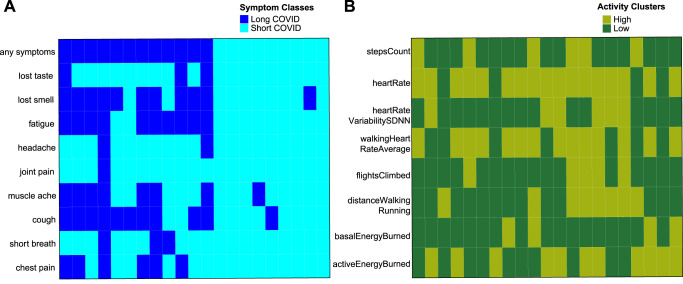
Heatmaps of unsupervised classes of 21 patients that have complete symptom and activity data. **A** This heatmap shows the binary symptom classes with cyan denoting patients classified in short COVID-19 profiles and dark blue denoting patients with a long COVID-19 symptom profile. **B** This heatmap shows the classification of individuals as high (light green) and low (dark green) activity clusters, however, please note that there is insignificant evidence of separate clusters for walkingHeartRateAverage.

**Fig. 6:**
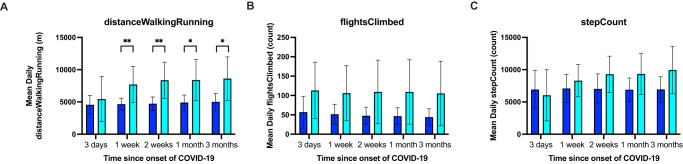
Personal activity following onset of COVID-19. Bar charts show mean with standard error baseline activities of short/long COVID-19 trajectories for **A** distanceWalkingRunning, **B** flightClimbed, **C** stepsCount. *p < 0.05, **p < 0.01 after adjustment for age, gender and comorbidities. *n* = 12 participants with Long COVID-19 and 9 participants with Short COVID-19.

There were 21 participants with both COVID-19 symptoms and longitudinal measurement of 8 physical activity patterns (Fig. 5, Supplementary Tables 9 and 10). Overall, there were weak evidence for association between the activity patterns and long- or short-COVID-19 symptoms. However, we observed a significant association between short COVID-19 and high activity patterns of distanceWalkingRunning (Table 2; Supplementary Table 11), accounting for age, gender, and comorbidities. Conversely, individuals in the long symptoms group had lower mean activity level for distanceWalkingRunning (Supplementary Table 12). To determine how early we can see this association, we measured the average activity of distanceWalkingRunning over windows of 3 days, 1 week, 2 weeks, 1 month and 3 months from COVID-19 onset. Individuals in the long COVID-19 symptoms cluster had a significantly lower mean activity level for distanceWalkingRunning compared to the short symptoms cluster starting from 1 week after disease onset (Fig. 6A). Other related activities, such as stepCount and flightsClimbed, were not associated in both the baseline and trajectory pattern measures (Fig. 6B, C).

**Table 2:** Association between long and short COVID-19 trajectories with low and high activity clusters.

Low vs high activity cluster	Low vs high activity cluster	
	Odds ratio	Fisher’s Exact test *p*-value
stepCount (number of steps taken)	0.68	1
heartrate (average heart rate)	1.40	1
heartRateVariabilitySDNN (standard deviation of heartbeat intervals)	0.68	1
walkingHeartRateAverage (average heart rate while walking)	1	1
flightsClimbed (number of flights of stairs climbed)	0.13	0.12
distanceWalkingRunning (distance moved walking or running)	0.12	0.03
basalEnergyBurned (resting energy burned)	0.71	1
activeEnergyBurned (active energy burned)	0.38	0.39

## Discussion

In this study, we followed 204 healthcare workers of whom 121 had COVID-19 symptoms tracked for over a year. From this cohort, 140 participants were recruited into the MyHeart Counts App Study. 54 participants owned or were provided with an Apple Watch Series 4 to measure physical activity. There was sufficient activity data for trajectory monitoring from 34 participants, 21 of whom reported symptoms of COVID-19. Unsupervised clustering of symptoms trajectories indicated the presence of long-term patterns in lost taste, lost smell, fatigue, headache, joint pain, muscle ache, cough, shortness of breath and chest pain. While the prevalence of any long-term symptoms was 36.3%, the most frequent long-term symptoms were fatigue (24.79%), lost smell (21.49%), chest pain (19.84%), and shortness of breath (18.18%). Eight physical activity features were analysed to identify two independent trajectories of high and low activity with distance walking or running being associated with short/long COVID-19 symptoms. For our final aim, we studied the association of the COVID-19 symptoms trajectory with the eight physical activity trajectory patterns. We only found a significant association between higher levels of distanceWalkingRunning with short COVID-19 trajectories. We reconfirmed this result by considering only the mean value of physical activity variables for different durations from COVID-19 onset. Despite the small sample size, this association stands even when we treat physical activity as two distinct clusters or just the mean values of physical activity in the long and short COVID-19 symptoms patterns. It is unclear why only distanceWalkingRunning was significantly associated but this may reflect that the data are collected during a ‘Workout’ with a higher frequency of data collection and/or the combination of effort and distance compared to e.g. stepCount.

The frequency of long symptoms in our cohort compared well with previous studies^4^, however, there is still variability (5–50%) in the estimated prevalence reported in those studies. This uncertainty may be due to the difference in definitions of duration, self-reporting of symptoms and also the number and type of symptoms considered in the cohort studies^6^. Higher risk for severe COVID-19 has previously been associated with physical inactivity^21^ and the importance of physical activity^13^ to reduce the severity of COVID-19 is emerging^22^. Previous studies have highlighted the potential for HRV to predict survival in hospitalised patients with COVID-19^8^ and there has been heightened interest in the use of wearable devices and smartphones to capture this data during the pandemic^11^. HRV significantly predicts survival rate and the percentage of referral to ICU within an early stage in hospitalised patients with COVID-19^15^.

Wearable devices may also detect variation in physical activity from the baseline due to infection. Participants with available activity data within a time span of 30 days before and after the estimated COVID-19 start date were analysed to calculate the mean activity per activity measure before and after SARS-CoV-2 infection. For each activity, the mean trajectory (Supplementary Fig. 6) was calculated for all participants with sufficient data and then we compared the mean pre/post COVID-19 activity level using a paired sample Wilcoxon singed ranked test. Despite the small sample size, we found that heartRateVariabilitySDNN, activeEnergyBurned, basalEnergyBurned and distanceWalkingRunning were different after SARS-CoV-2 infection. However, we had insufficient sample size to compare high and low activity trajectories before and after SARS-CoV-2 infection. Mason et al. 2022 developed a machine learning classifier that uses resting heart rate (RHR), heart rate variability (HRV), respiratory rate (RR), and dermal temperature from the Oura ring sensor to detect COVID-19 onset 2.75 days before participants realised they have COVID-19^23^, and similar to our observations they found a consistent pattern of HRV for up to 6 months after COVID-19 which could be differentiated between high and low activity groups.

The primary limitation of our study is the availability of frequent of COVID-19 testing during the first wave of the pandemic beginning March 2020. Many participants (asymptomatic or mild at infection) were identified by serological assay many weeks after they may have been infected, and even those who had reported symptoms and were asked to recall their symptoms weeks or months prior, which may have led to errors in reporting. However, previous studies have shown that there can be good agreement between self-reported data and medical records for the absence or presence of some respiratory conditions^24^. Unfortunately, routine molecular testing for influenza and other respiratory viruses is not performed in healthcare workers in the UK as standard practice. While it is impossible to rule out other co-infections during the study period, we are confident that our findings reflect the effects of COVID-19 due to several factors. Firstly, respiratory virus surveillance in the UK during this period of the COVID-19 pandemic demonstrated a dramatic reduction in circulation of most respiratory viruses other than SARS-CoV-2 because of non-pharmaceutical interventions^25^. Since our cohort groups were defined not only on a documented history of positive SARS-CoV-2 RT-PCR in symptomatic individuals, but also on seropositivity or seronegative on study entry using a SARS-CoV-2 spike and nucleocapsid assay demonstrated to have >99.5% sensitivity and specificity^26^ we are confident that the symptoms reported are due to COVID-19.

Secondly, as a cohort of HCWs, the participants were not permitted to wear a wristwatch during working hours due to infection control. This significantly limits the ability to collect the full range of heart rate data obtained via the watch PPG sensors, but metrics such as step counts, and distance walked can still be obtained via the paired smartphone in the participant’s pocket. As a UK HCW study, our population was skewed to a higher percentage of female participants, and perhaps younger than some other cohorts, particularly compared to those with hospitalised COVID-19 cases. Finally, there is a substantial discrepancy in the number of devices supplied and the amount of data captured from participants. There are several well described examples of how data is lost to follow-up events in remote monitoring studies with consumer devices and smartphone applications^20^. These include a wide spectrum of reasons including but not limited to people forgetting to wear their devices, not properly setting up the app, and not syncing or sharing data. In this work, we demonstrate the use of Fretchet distance-based clustering for physical activity parameters time series classification measured from wearables. For symptoms trajectories, we imputed and clustered using Generalized Linear Models and Latent Class Growth Analysis on a mixture of clinically reported and self-reported data. Other methods such as DBscan^27^ based on spatial densities of points can be utilized for clustering on larger data sizes.

Despite the limitations and the relatively small cohort, we have demonstrated an approach to apply unsupervised trajectory analysis that identified significantly different clusters of both COVID-19 symptoms and physical activity that suggest a weak association between the length of reported COVID-19 symptoms and activity could be further interrogated. This study also highlights the potential value of routinely collecting data from consumer wearables, and the challenges of integrating and interpreting these data as part of a healthcare record.

## Methods

### Participant recruitment and COVID-19 status

All participants (over 18 years of age) were recruited into the Sheffield Teaching Hospitals NHS Foundation Trust Observational study of pulmonary hypertension, cardiovascular and respiratory diseases study (STH-ObS, 18/YH/0441) following informed written consent (Table 1). Eligible participants currently working as healthcare workers, including allied support and laboratory staff, and were in possession of an Apple iPhone 6 or later were offered an Apple Watch Series 4 as part of the study. No participants were hospitalised for COVID-19 throughout the duration of the study. Procedures were done in compliance with the principles of the Declaration of Helsinki (2008) and the International Conference on Harmonisation Good Clinical Practice guidelines.

### MyHeart Counts smartphone app and data linkage to COVID-19 clinic records

MyHeart Counts is a smartphone app that works on the Apple iPhone to collect information about an individual’s cardiovascular health, wellbeing, diet, smoking history and can perform a six-minute walking test following in-app electronic consent^20,28^. In addition to these self-reported questionnaires, the App can pull heart rate data from compatible wearables through Apple HealthKit and has been used as a platform for a randomised control interventional study of physical activity^29^. Descriptors of HealthKit data types is available at https://developer.apple.com/documentation/healthkit/hkquantitytypeidentifier. During the early stage of the COVID-19 pandemic MyHeart Counts was updated (Version 2.3.0) to include self-reported COVID-19 symptoms and testing. Each participant recruited into STH-ObS who met the study criteria was provided with a pseudonymous identifier and instruction on how to enter this into the App. This pseudonymous identifier was then used to link HealthKit data obtained from the App to their clinical record collected through research clinics. For each study participant, the time of diagnosis was determined by the date of first reported positive PCR test or date of positive serology test, whichever came earlier. For the physical activity data, the start of the time series analysis was determined by the date of first activity. 20 participants were excluded from the study analysis due to insufficient activity data. Lack of data was determined based on either the complete absence of activity data around the participant’s time of diagnosis or having less than 30 time-points/days.

### Modelling COVID-19 symptoms trajectories

The aim of this analysis was to identify distinct data-driven unsupervised longitudinal trends in symptoms, and physical activity, and subsequently test the association between them. The data structure of symptoms data and the physical activity data was different. We therefore took two different approaches to identify the underlying data-driven trends within each dataset. To identify patterns of reported COVID-19 symptoms over time, analysis was performed with at least two timepoints of symptom observations, including at the first report of a COVID-19 positive test. We used a generalised linear model with probit link function (‘glmfit’ model in Statistical and Machine learning Toolbox, Matlab version 2017a) to obtain the fitted model coefficients. The output of this model is an estimate of the number of positive symptoms corresponding to the number of symptoms assessed as a function of time. Symptom data consisted of two types of assessments; (i) from the clinical visits (17 symptoms) and (ii) from self-reporting via the MyHeart Counts app (10 symptoms). Since each assessment had a different number of symptom questions, we estimated the ratio between the number of positive symptoms (successes) and the total number of symptoms assessed at each timepoint, calculated as: $P=\frac{{{\rm{Number}}\; {\rm{of}}\; {\rm{positive}}\; {\rm{symptoms}}}}{{{\rm{Number}}\; {\rm{of}}\; {\rm{symptoms}}\; {\rm{assessed}}}\,({Nq})}$. This was used to establish the success percent, or the probability of success out of Nq trials. The number of symptoms assessed *Nq* was equal to 17 during clinical visits and equal to 10 for self-reported symptoms via the app.

To derive unsupervised longitudinal patterns using Latent Class growth analysis (LCGA) using MPLUS^29^, we required all subjects’ trajectories to be measured at common timepoints. Therefore, we estimated the probability of the presence of any symptom at five equally spaced common timepoints over a year as: [0, 90, 180, 270, 360] days. The estimated probabilities at these timepoints were then used as inputs to LCGA to derive unsupervised longitudinal patterns via the criterion of bootstrapped log-likelihood ratio test *p*-value < 0.05, high entropy for classification, high average posterior probability of belonging to a class, studying the linear and quadratic growth patterns, successful convergence (best log-likelihood ratio estimates were repeated)^30,31^ and investigating the 95% confidence intervals of the observed mean patterns of each classified group. On similar lines, we applied the Generalized Linear Model with probit link function with *Nq* = 1 to get the probability of presence of an individual symptom at common timepoints of [0, 90, 180, 270, 360] days to derive patterns of classification in each specific symptom.

We performed a sensitivity analysis to test the effect of changing both the timepoints and the total duration over which the data were collected on the classification performance. To test whether our sample size was sufficient, we examined how serial down-sampling of the participants affected this method’s ability to identify distinct clusters.

### Clustering of physical activity trajectories

Of the recruited individuals using the MyHeart Counts App with an Apple Watch there was sufficient longitudinal data for a time-series analysis on eight HealthKit activity measures; basalEnergyBuned (resting energy burned by the individual) activeEnergyBurned (active energy burned by the individual), flightsClimbed (number flights of stairs that the individual has climbed), distanceWalkingRunning (distance the individual has moved by walking or running), heartrate (individuals average heart rate), walkingHeartRateAverage (individuals average heart rate while walking), heartRateVariabilitySDNN (standard deviation of heartbeat intervals of the individual), stepCount (number of steps the individual has taken) from 34 individuals. Of these, walkingHeartRateAverage, flightsClimbed, and stepCount were the most frequent activities recorded with on average 496.5, 493.5 and 493.5 timepoints, respectively, and basalEnergyBurned recorded the least (472 timepoints). Data from these individuals was imported to R (version 4.2.0) and filtered based on the availability of relevant dates. Trajectories of 34 individuals were created depending on the specific activity and varied in number of timepoints ranging between 42 and 596. We hypothesised that while everyone’s physical activity profile over time may be slightly different, we would see common patterns of activity across multiple individuals, especially after COVID-19 infection. To identify distinct groups of individuals with similar longitudinal patterns of physical activity, we utilised a shaped-based approach for clustering. The trajectories were analysed using the longitudinal clustering KmlShape R package v0.9.5 to generate activity profiles for each variable to reduce the complexity and computational requirements of longitudinal clustering as required by the algorithm. The trajectories were first minimally merged based on the Fretchet distance^32^ by calculating the distance between each pair of trajectories and subsequently using it to generate 30–33 senator trajectories. The senator trajectories represent the actual trajectories but reduce the number of calculations needed for the following clustering analysis. In turn, the senator trajectories were reduced to 100 timepoints for the purposes of calculating shape similarities. Multiple clustering analyses were carried out with selected k number of clusters ranging between two and five to create activity profiles for every type of activity measured. Two-cluster outputs were most stable for each activity. The differences between the mean representative trajectories of each cluster were calculated by averaging the trajectories of their members and smoothed by local polynomial regression fitting (loess in R Stats package v 4.2.1). To calculate the differences between low and high cluster trajectories we used a Welch Two Sample t-test between the means of the two activity clusters. Each activity cluster was represented by a senator trajectory (calculated based on a classical k-means algorithm in kmlShape R package 0.9.5) which encapsulates the mean curve of the patient cluster members.

### Association testing between physical activity and symptom trajectories

We tested for an association between each activity trajectory COVID-19 symptom trajectory by building a contingency table for each using observed and expected values and performing a Fisher’s Exact test. We also employed a two-sample t-test to check for univariable associations of physical activity against symptom group. The activity level was calculated at single time ranges from onset of symptoms (averaged from day 1–3 days, 1 week, 2 weeks, 1 month and 3 months after onset and confirmation of diagnosis) in each COVID-19 symptom trajectory classification. For baseline physical activity only data between the 5th and 95th percentiles were used to avoid extreme outlier values.

### Supplementary information

Supplemental Material

## Data Availability

Data relating to COVID-19 symptoms and clinical features are available from the corresponding authors via MTAs with the Sheffield Teaching Hospitals observational study of pulmonary hypertension, cardiovascular and other respiratory diseases (STH-ObS, UK REC Ref 18/YH/0441). Pseudo-anonymised activity data are also available for cases who have been agreed to external data sharing.

## Code availability

Scripts for the analysis of symptom trajectories are available form https://github.com/BioSok/covid19_symptoms_activity_trajectories, and scripts for the analysis of physical activity data are available from https://github.com/AshleyLab/myheartcounts.
