# Supplementary material for: Unsupervised machine learning to investigate trajectory patterns of COVID-19 symptoms and physical activity measured via the MyHeart Counts App and smart devices
Source: NPJ Digit Med. 2023 Dec 22;6:239. doi: 10.1038/s41746-023-00974-w (PMC10746711; doi:10.1038/s41746-023-00974-w)
Supplement: Supplementary file 1 — Supplemental Material [file 41746_2023_974_MOESM1_ESM.docx]

**Supplementary Materials**

**Supplementary Table 1. Underlying medical conditions reported by more than 2 participants.**

| **Underlying Conditions** | **Number Affected** |
| --- | --- |
| Acute asthma | 14 |
| Autoimmune hypothyroidism | 13 |
| Benign essential hypertension | 9 |
| Mild intermittent asthma | 6 |
| Diabetes mellitus | 6 |
| Migraine | 5 |
| Celiac disease | 3 |
| Gastroesophageal reflux disease | 3 |

**Supplementary Table 2. Entropy and posterior probability of classes corresponding to each classified symptom.**

| Symptom | Number (percentage) of patients in Long/Short COVID | Entropy | Posterior Probability  (Long/Short COVID) |
| --- | --- | --- | --- |
| Any symptom | 44/77 | 0.98 | 0.99/1.00 |
| Lost Taste | 12/109 | 1.00 | 1.00/1.00 |
| Lost Smell | 26/95 | 1.00 | 1.00/1.00 |
| Fatigue | 30/91 | 1.00 | 1.00/1.00 |
| Headache | 12/109 | 1.00 | 1.00/1.00 |
| Joint pain | 9/112 | 1.00 | 1.00/1.00 |
| Muscle Ache | 19/102 | 1.00 | 1.00/1.00 |
| Cough | 20/101 | 0.99 | 1.00/1.00 |
| Short Breath | 21/100 | 0.79 | 0.97/0.95 |
| Chest pain | 24/97 | 1.00 | 1.00/1.00 |


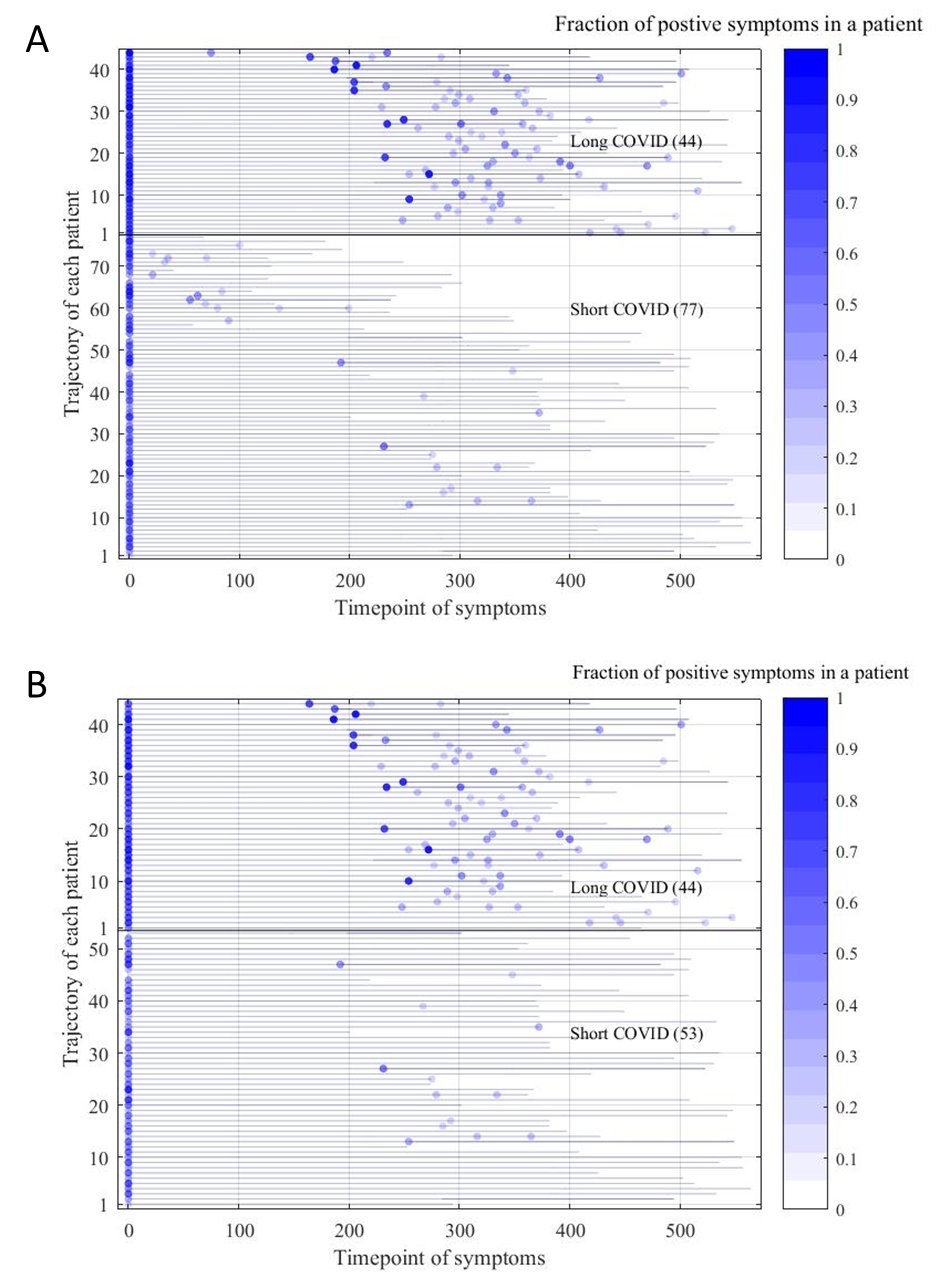


**Supplementary Figure 1. Symptom timelines for each patient.** A) Long and short trajectories corresponding to any symptoms trajectories for 121 subjects. B) Sensitivity analysis to show classification of 97 subjects trajectories with time point of baseline before July 1,2020 which consisted mainly of short trajectories. Classification of long trajectories remained robust to removal of some of the short trajectories.


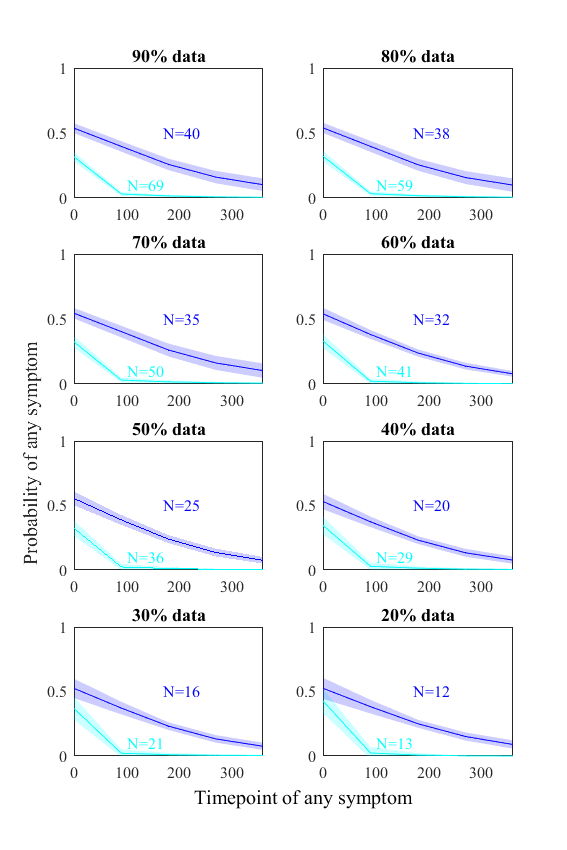


**Supplementary Figure 2. Effects of down-sampling of symptoms data (by randomly removing10% of the samples) on classification patterns of any symptom.** The 95% confidence region around the mean trajectory patterns start overlapping when sample size reduces to <40% of the original sample size, N represents the number of trajectories in each class.

**
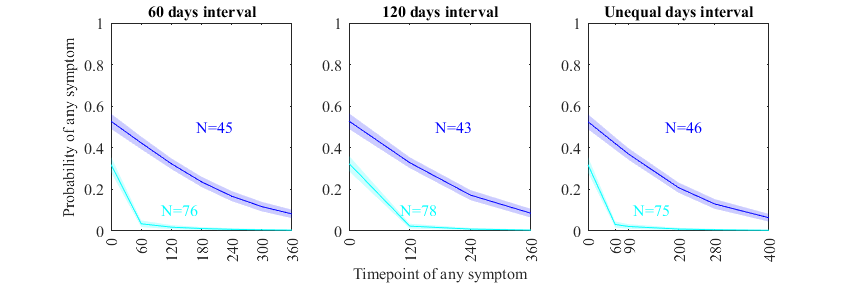
**

**Supplementary Figure 3. Trajectory patterns of any symptom derived using different timepoints and duration of symptoms probability estimation.** Shaded regions are the 95% confidence interval around mean trajectory patterns and N represents the number of trajectories in each class.

**Supplementary Table 3. Inter-rater agreement of symptoms trajectory classification using different timepoints and duration of symptoms probability estimation.**

| Interval | Symptoms trajectory class | 90 days interval | | | Cohen's kappa |
| --- | --- | --- | --- | --- | --- |
|  |  | 77 | 44 | total |  |
|  |  | short | long |  |  |
| 60 days | short | 76 | 0 | 76 | 0.98 |
|  | long | 1 | 44 | 45 |  |
| 120 days | short | 77 | 1 | 78 | 0.98 |
|  | long | 0 | 43 | 43 |  |
| Unequal | short | 75 | 0 | 75 | 0.97 |
|  | long | 2 | 44 | 46 |  |


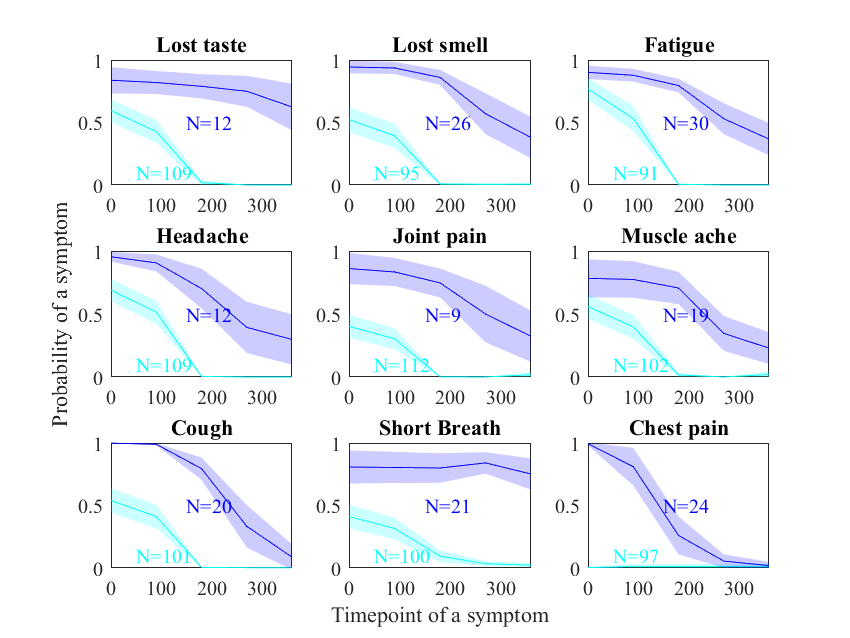


**Supplementary Figure 4. Extrapolated probabilities of presence of an individual symptom.** 95% confidence interval for individual symptoms at five common timepoints [0 90 180 270 360 days] for long and short trajectory groups derived from unsupervised clustering.

**Supplementary Table 4. Prevalence of long-term COVID-19 symptoms in other studies compared to this study.**

| Study | Any symptom | Loss of taste | loss of smell | Fatigue | Headache | Joint pain | Muscle ache | cough | short breath | chest pain |
| --- | --- | --- | --- | --- | --- | --- | --- | --- | --- | --- |
| Current work mean[95% confidence]  (median follow-up = 408 [41 - 564] days) | 36.4  [26.4, 48.8]% | 9.9  [5.1, 17.3]% | 21.5  [14.0, 31.5]% | 24.8[ 16.7, 35.4]% | 9.9  [5.10, 17.3]% | 7.4  [3.4,14.1]% | 15.7  [9.5, 24.5]% | 16.5 [10.1, 25.5]% | 17.36 [13.10, 32.10]% | 19.8  [12.7, 29.5]% |
| Boscolo-Rizzo et al. 2022  (730 days)^1^ | 28.0% | 8.3% | | 18.5% |  |  |  |  | 10.7% |  |
| Peterson et al 2022  (168 days) ^2^ | 39.0% | 14.0% | 17.0% | 17.0% | 6.5% | 2.4% | 2.0% | 3.0% | 7.0% | 4.0% |
| Pérez‑González et al. 2022  (183 days) ^3^ | 48.9% | 4.0% | 6.9% | 16.1% | 4.8% | 4.8% | 4.0% | 4.4% | 20.6% | 6.0% |
| Chen et al. 2022  (120 days) ^4^ | 49.0%  (120 days) | 8.0% | 7.0% | 23.0% | 5.0% | 10.0% | 6.0% | 7.0% | 13.0% | 5.0% |
| Cortinovis et al. 2021  (183 days, hospitalized) ^5^ | 76.0% |  |  | 63.0%  (fatigue or weakness) |  |  |  |  |  |  |
| Michelen et al. 2021  (84 days or more) ^6^ |  | 13.5% | 15.2% | 31.0% | 4.9% | 9.4% | 11.3% | 8.2% | 25.1% | 6.4% |
| Lopez-Leon et al. 2021  (14-110 days) ^7^ | 80.0% | 23.0% | 21.0% | 58.0% | 44.0% | 19.0% |  | 19.0% | 24.0% | 16.0%  (chest discomfort) |

**Supplementary Table 5. Odds of different parameters associated with long trajectory patterns of any or specific symptoms.** Values with NaN indicates that there were not enough cases to compute the odds.

|  | **Any symptoms** | | | | **Lost taste** | | | |
| --- | --- | --- | --- | --- | --- | --- | --- | --- |
| *Variable* | *Odds ratio* | *5% CI* | *95% CI* | *p-value* | *Odds ratio* | *5% CI* | *95% CI* | *p-value* |
| number_symptoms_baseline | 1.95 | 1.51 | 2.50 | 0.00 | 1.41 | 1.09 | 1.83 | 0.01 |
| has_comorbidity[T.True] | 1.23 | 0.45 | 3.34 | 0.69 | 0.78 | 0.21 | 2.96 | 0.72 |
| gender[T.Male] | 0.52 | 0.12 | 2.36 | 0.40 | 0.42 | 0.09 | 2.05 | 0.28 |
| ethnicity[T.White British] | 1.17 | 0.27 | 5.16 | 0.83 | 2.19 | 0.41 | 11.8 | 0.36 |
| above_44yrs[T.Yes] | 1.24 | 0.46 | 3.33 | 0.66 | 0.92 | 0.24 | 3.51 | 0.91 |
|  | **Lost smell** | | | | **Fatigue** | | | |
| *Variable* | *Odds ratio* | *5% CI* | *95% CI* | *p-value* | *Odds ratio* | *5% CI* | *95% CI* | *p-value* |
| number_symptoms_baseline | 1.40 | 1.16 | 1.69 | 0.00 | 1.49 | 1.23 | 1.81 | 0.00 |
| has_comorbidity[T.True] | 1.35 | 0.51 | 3.59 | 0.55 | 0.29 | 0.10 | 0.79 | 0.02 |
| gender[T.Male] | 0.76 | 0.19 | 3.07 | 0.69 | 0.71 | 0.15 | 3.27 | 0.66 |
| ethnicity[T.White British] | 0.94 | 0.21 | 4.24 | 0.94 | 0.38 | 0.08 | 1.89 | 0.24 |
| above_44yrs[T.Yes] | 1.43 | 0.54 | 3.76 | 0.47 | 0.75 | 0.28 | 1.99 | 0.56 |
|  | **Headache** | | | | **Joint pain** | | | |
| *Variable* | *Odds ratio* | *5% CI* | *95% CI* | *p-value* | *Odds ratio* | *5% CI* | *95% CI* | *p-value* |
| number_symptoms_baseline | 1.15 | 0.92 | 1.42 | 0.22 | 1.44 | 1.04 | 1.97 | 0.03 |
| has_comorbidity[T.True] | 0.91 | 0.24 | 3.43 | 0.88 | 0.44 | 0.07 | 2.70 | 0.38 |
| gender[T.Male] | NaN | NaN | NaN | NaN | NaN | NaN | NaN | NaN |
| ethnicity[T.White British] | 2.16 | 0.37 | 12.8 | 0.39 | 3.02 | 0.41 | 22.1 | 0.28 |
| above_44yrs[T.Yes] | 0.77 | 0.20 | 2.96 | 0.71 | 2.46 | 0.48 | 12.7 | 0.28 |
|  | **Muscle ache** | | | | **Cough** | | | |
| *Variable* | *Odds ratio* | *5% CI* | *95% CI* | *p-value* | *Odds ratio* | *5% CI* | *95% CI* | *p-value* |
| number_symptoms_baseline | 1.48 | 1.17 | 1.86 | 0.00 | 1.62 | 1.26 | 2.09 | 0.00 |
| has_comorbidity[T.True] | 0.63 | 0.19 | 2.05 | 0.44 | 1.08 | 0.34 | 3.46 | 0.90 |
| gender[T.Male] | NaN | NaN | NaN | NaN | 5.14 | 0.56 | 47.0 | 0.15 |
| ethnicity[T.White British] | 1.75 | 0.33 | 9.36 | 0.51 | 3.48 | 0.70 | 17.4 | 0.13 |
| above_44yrs[T.Yes] | 2.23 | 0.72 | 6.92 | 0.17 | 1.54 | 0.50 | 4.73 | 0.45 |
|  | **Short breath** | | | | **Chest pain** | | | |
| *Variable* | *Odds ratio* | *5% CI* | *95% CI* | *p-value* | *Odds ratio* | *5% CI* | *95% CI* | *p-value* |
| number_symptoms_baseline | 1.29 | 1.07 | 1.56 | 0.01 | 1.62 | 1.26 | 2.09 | 0.00 |
| has_comorbidity[T.True] | 1.71 | 0.59 | 4.93 | 0.32 | 1.08 | 0.34 | 3.46 | 0.90 |
| gender[T.Male] | 0.48 | 0.12 | 1.96 | 0.31 | 5.14 | 0.56 | 47.0 | 0.15 |
| ethnicity[T.White British] | 0.73 | 0.13 | 4.12 | 0.72 | 3.48 | 0.70 | 17.4 | 0.13 |
| above_44yrs[T.Yes] | 1.37 | 0.48 | 3.90 | 0.56 | 1.54 | 0.50 | 4.73 | 0.45 |

**Supplementary Table 6. Odds of patients getting reinfected with a particular symptom after more than 1 year from first infection for individuals with long symptom trajectories compared to short trajectories.** Odds were calculated for the 38 patients who got reinfected by any of the symptoms. For cases with zero counts, we applied Haldane's correction.

|  | Long COVID (14) | | Short COVID(24) | | OddsRatio(95% confidence) |
| --- | --- | --- | --- | --- | --- |
| Reinfection | yes | no | yes | no |  |
| **Nausea/vomit** | 4 | 10 | 0 | 24 | **21.0 (1.04,426)** |
| Abdominal pain | 3 | 11 | 0 | 24 | 14.9 (0.710,313) |
| **Joint pain** | 5 | 9 | 1 | 23 | **12.8(1.31,125.0)** |
| **Muscle ache** | 10 | 4 | 7 | 17 | **6.07(1.42,26.03)** |
| Lost taste | 2 | 12 | 1 | 23 | 3.83(0.315,46.7) |
| Headache | 9 | 5 | 8 | 16 | 3.60(0.902,14.4) |
| Sore throat | 11 | 3 | 13 | 11 | 3.10(0.687,14.0) |
| Runny nose | 9 | 5 | 10 | 14 | 2.52 (0.646,9.83) |
| fatigue | 10 | 4 | 13 | 11 | 2.12(0.516, 8.67) |
| Diarrhoea | 2 | 12 | 2 | 22 | 1.83(0.229,14.7) |
| Short breath | 1 | 13 | 1 | 23 | 1.77(0.102, 30.7) |
| Confusion | 0 | 14 | 0 | 24 | 1.69(0.0318, 89.8) |
| Chest pain | 0 | 14 | 0 | 24 | 1.69 (0.0318, 89.8) |
| Wheeze | 0 | 14 | 0 | 24 | 1.69 (0.0318, 89.8) |
| Cough | 6 | 8 | 8 | 16 | 1.50(0.386, 5.83) |
| Lost smell | 2 | 12 | 3 | 21 | 1.17 (0.170,8.00) |
| Fever | 5 | 9 | 9 | 15 | 0.926(0.235, 3.65) |

**Supplementary Table 7. Timepoint statistics and details for the clusters generated for each HealthKit activity variable**

| **HealthKit Activity variables** | **N** | **Min** | **Mean** | **Median** | **Max** |
| --- | --- | --- | --- | --- | --- |
| heartRate (count/second) | 33 | 56.0 | 414.4 | 487.0 | 593.0 |
| basalEnergyBurned (calories) | 34 | 75.0 | 406.3 | 472.0 | 593.0 |
| stepCount  (count) | 32 | 67.0 | 421.3 | 493.5 | 593.0 |
| heartRateVariability  (millisecond) | 32 | 87.0 | 429.2 | 489.0 | 593.0 |
| flightsClimbed  (count) | 32 | 88.0 | 434.5 | 496.5 | 596.0 |
| distanceWalkingRunning  (meters) | 31 | 42.0 | 411.1 | 487.0 | 593.0 |
| walkingHeartRateAverage  (count/second) | 32 | 86.0 | 428.2 | 493.5 | 593.0 |
| activeEnergyBurned (calories) | 34 | 62.0 | 426.6 | 481.0 | 593.0 |

**Supplementary Table 8. Longitudinal activity clustering.** The clusters formed by longitudinal clustering for each of the 8 physical activity measures along with their sizes and the statistical significance of the difference between the mean activity level of each cluster. Significant differences between low and high cluster trajectories calculated using Welch Two Sample t-test between the means of each activity cluster was used. Each activity cluster was represented by a senator trajectory which encapsulates the mean trajectory curve of the patient cluster members (calculated based on a classical k-means algorithm in kmlShape R package 0.9.5).

| **HealthKit Activity variables** | **Cluster 1 (low activity)** | | **Cluster 2 (high activity)** | | **p-value**  **(mean_c1-c2_)** |
| --- | --- | --- | --- | --- | --- |
|  | **n** | **Trajectory Mean**  **(min/max)** | **n** | **Trajectory Mean**  **(min/max)** |  |
| **heartRate**  **(count/second)** | 25 | 1.4  (0.8-2.7) | 8 | 1.42  (0.8-2.7) | 2.9e-07 |
| **basalEnergyBurned**  **(calories)** | 25 | 1,716,256.0  (14,896.0-32,318,588.0) | 9 | 2,251,475.0  (14,979.0-63,452,416.0) | 2.2e-16 |
| **stepCount**  **(count)** | 21 | 6763.2  (4.0-64,102.0) | 11 | 11,753.8  (2.0-218,935.0) | 2.2e-16 |
| **heartRateVariabilitySDNN**  **(millisecond)** | 24 | 35.9  (7.8-164.0) | 8 | 60.6  (14.1-212.7) | 2.2e-16 |
| **flightsClimbed**  **(count)** | 28 | 61.1  (1.0-489.0) | 4 | 304.2  (2.0-1,062.0) | 2.2e-16 |
| **distanceWalkingRunning**  **(meters)** | 18 | 4964.5  (1.3-43,860.9) | 13 | 10,955.7  (1.51-481,780.4) | 2.2e-16 |
| **walkingHeartRateAverage**  **(count/second)** | 19 | 1.71  (0.8-2.9) | 13 | 1.7  (0.9-3.0) | 0.359 |
| **activeEnergyBurned**  **(calories)** | 27 | 538,190.8  (21.0-5,267,592.0) | 7 | 949,650.0  (37.0-25,279,771.0) | 2.2e-16 |


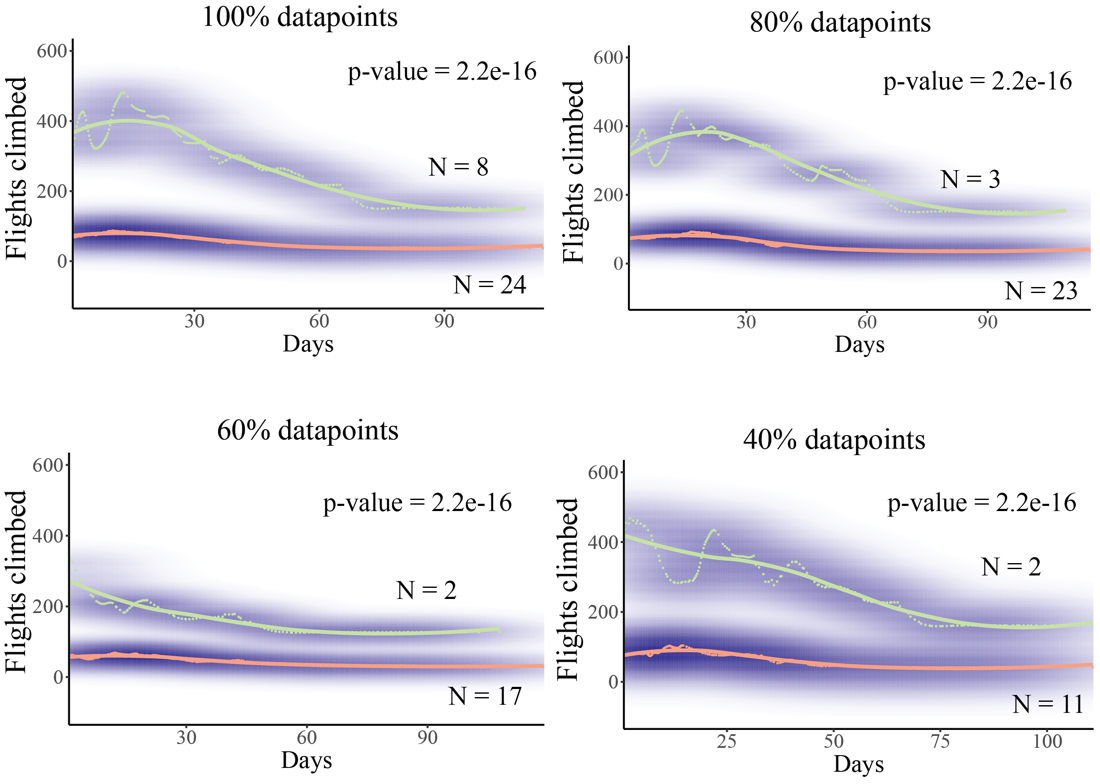


**Supplementary Figure 5. Effects of down-sampling patients used of activity clusters.** The relative percentage of patients whose physical data (example using flights climbed) on stability and significance of high and low activity cluster trajectories.

**Supplementary Table 9. Distribution of long and short symptoms trajectory cases across individuals (n=21).**

| **Symptom** | **Long Covid** | **Short Covid** |
| --- | --- | --- |
| Any symptom | 12 | 9 |
| Lost taste | 3 | 18 |
| Lost smell | 11 | 10 |
| fatigue | 10 | 11 |
| headache | 2 | 19 |
| Joint pain | 1 | 20 |
| Muscle ache | 8 | 13 |
| cough | 11 | 10 |
| Short breath | 3 | 18 |
| Chest pain | 6 | 15 |

**Supplementary Table 10. Distribution of low and high activity trajectory cases across individuals (n=21).**

| **HealthKit Activity Variables** | **Low** | **High** |
| --- | --- | --- |
| heartRate  (count/second) | 4 | 17 |
| basalEnergyBurned  (calories) | 17 | 4 |
| stepCount  (count) | 15 | 6 |
| heartRateVariabilitySDNN  (millisecond) | 15 | 6 |
| flightsClimbed  (count) | 16 | 5 |
| distanceWalkingRunning  (meters) | 13 | 8 |
| walkingHeartRateAverage  (count/second) | 7 | 14 |
| activeEnergyBurned  (calories) | 10 | 11 |

**Supplementary Table 11.** Contingency table for high/low activity and long/short covid used to compute Chi-squared tests of independence in Table 2. Expected values are computed under the assumption that symptom and activity clusters are independent.

| **HealthKit Activity Variables** | **Observed Values** | | **Expected Values** | |
| --- | --- | --- | --- | --- |
| **stepCount** | | | | |
|  | High Activity | Low Activity | High Activity | Low Activity |
| long_covid | 3 | 9 | 3.4 | 8.6 |
| short_covid | 3 | 6 | 2.6 | 6.4 |
| **heartRate** | | | | |
|  | High Activity | Low Activity | High Activity | Low Activity |
| long_covid | 10 | 2 | 9.7 | 2.3 |
| short_covid | 7 | 2 | 7.3 | 1.7 |
| **heartRateVariabilitySDNN** | | | | |
|  | High SDNN | Low SDNN | High SDNN | Low SDNN |
| long_covid | 3 | 9 | 3.4 | 8.6 |
| short_covid | 3 | 6 | 2.6 | 6.4 |
| **walkingHeartRateAverage** | | | | |
|  | High Activity | Low Activity | High Activity | Low Activity |
| long_covid | 8 | 4 | 8 | 4 |
| short_covid | 6 | 3 | 6 | 3 |
| **flightsClimbed** | | | | |
|  | High Activity | Low Activity | High Activity | Low Activity |
| long_covid | 1 | 11 | 2.9 | 9.1 |
| short_covid | 4 | 5 | 2.1 | 6.9 |
| **distanceWalkingRunning** | | | | |
|  | High activity | Low Activity | High Activity | Low Activity |
| long_covid | 2 | 10 | 4.6 | 7.4 |
| short_covid | 6 | 3 | 3.4 | 5.6 |
| **basalEnergyBurned** | | | | |
|  | High Activity | Low Activity | High Activity | Low Activity |
| long_covid | 2 | 10 | 2.3 | 9.7 |
| short_covid | 2 | 7 | 1.7 | 7.3 |
| **activeEnergyBurned** | | | | |
|  | High Activity | Low Activity | High Activity | Low Activity |
| long_covid | 5 | 7 | 6.3 | 5.7 |
| short_covid | 6 | 3 | 4.7 | 5.7 |

**Supplementary Table 12:** Association of short/long COVID-19 trajectories to mean levels of baseline activities over 3 days, 1 week, 2 weeks, 1 month and 3 months following onset of COVID.

| **Baseline HealthKit Activity** | **Long vs Short COVID Symptoms** | | | | | | | | | |
| --- | --- | --- | --- | --- | --- | --- | --- | --- | --- | --- |
|  | 3 days | | 1 week | | 2 week | | 1 month | | 3 months | |
|  | Mean difference | Adj P-val | Mean difference | Adj P-val | Mean difference | Adj P-val | Mean difference | Adj P-val | Mean difference | Adj P-val |
| BasalEnergyBurned (calories) | 36503 | 0.944 | 84146 | 0.870 | 100383 | 0.848 | 66783 | 0.887 | 211149 | 0.517 |
| DistanceWalkingRunning (meters) | 881 | 0.517 | 3040 | 0.018 * | 3628 | 0.008** | 3486 | 0.018 * | 3606 | 0.019 * |
| ActiveEnergyBurned (calories) | 38566 | 0.795 | 86283 | 0.589 | 56267 | 0.737 | 1526 | 0.991 | 15762 | 0.901 |
| FlightsClimbed (count) | 56 | 0.068 | 55 | 0.031 * | 61 | 0.047 * | 63 | 0.025 * | 61 | 0.031 * |
| HeartRate (count/second) | 0.015 | 0.840 | 0.054 | 0.554 | 0.130 | 0.141 | 0.110 | 0.166 | 0.070 | 0.386 |
| HeartRateVariabilitySDNN (millisecond) | 4.310 | 0.345 | 2.720 | 0.471 | 4.070 | 0.264 | 2.700 | 0.458 | 1.600 | 0.620 |
| StepCount (count) | 880 | 0.523 | 1215 | 0.264 | 2253 | 0.067 | 2438 | 0.042 * | 3120 | 0.017 * |
| WalkingHeartRateAverage (count/second) | 0.120 | 0.145 | 0.110 | 0.149 | 0.090 | 0.232 | 0.100 | 0.177 | 0.100 | 0.177 |

| distanceWalkingRunning | | |  |  |  |  |  |  |  |  |  |
| --- | --- | --- | --- | --- | --- | --- | --- | --- | --- | --- | --- |
| any_symp | count | 3 days | | 1 week | | 2 week | | 1 month | | \| 3 months \| \| --- \| | |
|  |  | mean | sd | mean | sd | mean | sd | mean | sd | mean | sd |
| long_covid | 12 | 4566 | 1439 | 4661 | 918 | 4737 | 1047 | 4910 | 1162 | 5017 | 1284 |
| short_covid | 8 | 5447 | 3497 | 7701 | 2791 | 8364 | 2815 | 8396 | 3208 | 8623 | 3375 |

| flightsClimbed | |  |  |  |  |  |  |  |  |  |  |
| --- | --- | --- | --- | --- | --- | --- | --- | --- | --- | --- | --- |
| any_symp | count | 3 days | | 1 week | | 2 week | | 1 month | | 3 months | |
|  |  | mean | sd | mean | sd | mean | sd | mean | sd | mean | sd |
| long_covid | 12 | 57 | 41.1 | 51.5 | 25.9 | 47.6 | 22.2 | 46.6 | 21.6 | 44 | 21.5 |
| short_covid | 8 | 113 | 73 | 106 | 71 | 109 | 82.3 | 109 | 83.3 | 105 | 83.3 |

| stepCount | |  |  |  |  |  |  |  |  |  |  |
| --- | --- | --- | --- | --- | --- | --- | --- | --- | --- | --- | --- |
| any_symp | count | 3 days | | 1 week | | 2 week | | 1 month | | 3 month | |
|  |  | mean | sd | mean | sd | mean | sd | mean | sd | mean | sd |
| long_covid | 12 | 6914 | 2081 | 7076 | 2187 | 7022 | 2330 | 6899 | 1804 | 6932 | 1974 |
| short_covid | 8 | 6034 | 3960 | 8291 | 2489 | 9275 | 2815 | 9337 | 3194 | 9948 | 3666 |


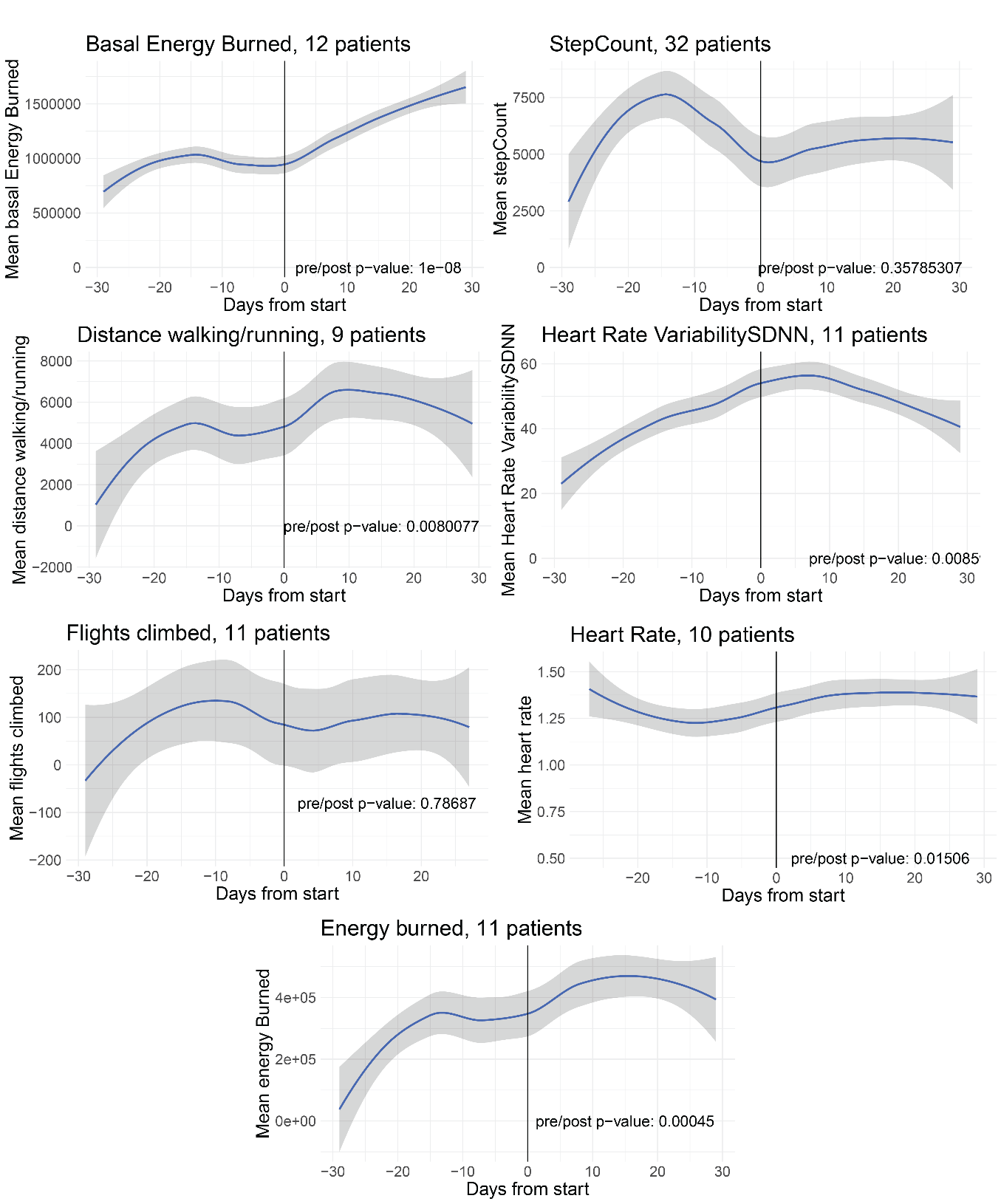


**Supplementary Figure 6. Mean trajectories of the activity measures pre and post COVID.** The mean trajectories of pre and post covid start date for each activity measure (as defined for the trajectory analysis). Windows of 30 days were considered for both cases and the number of patients included in the calculation of the means (i.e. with available data) is denoted on each plot. The p-values were calculated using a paired sample Wilcoxon singed ranked Test (function wilcox.test of R package stats 4.2.1).

**References (Supplemental Table 4)**

1 Boscolo-Rizzo, P. *et al.* Two-Year Prevalence and Recovery Rate of Altered Sense of Smell or Taste in Patients With Mildly Symptomatic COVID-19. *JAMA Otolaryngol Head Neck Surg* **148**, 889-891 (2022). https://doi.org:10.1001/jamaoto.2022.1983

2 Petersen, M. S. *et al.* Prevalence of long COVID in a national cohort: longitudinal measures from disease onset until 8 months' follow-up. *Int J Infect Dis* **122**, 437-441 (2022). https://doi.org:10.1016/j.ijid.2022.06.031

3 Perez-Gonzalez, A. *et al.* Author Correction: Long COVID in hospitalized and non-hospitalized patients in a large cohort in Northwest Spain, a prospective cohort study. *Sci Rep* **12**, 13873 (2022). https://doi.org:10.1038/s41598-022-18023-z

4 Chen, C. *et al.* Global Prevalence of Post-Coronavirus Disease 2019 (COVID-19) Condition or Long COVID: A Meta-Analysis and Systematic Review. *J Infect Dis* **226**, 1593-1607 (2022). https://doi.org:10.1093/infdis/jiac136

5 Cortinovis, M., Perico, N. & Remuzzi, G. Long-term follow-up of recovered patients with COVID-19. *Lancet* **397**, 173-175 (2021). https://doi.org:10.1016/S0140-6736(21)00039-8

6 Michelen, M. *et al.* Characterising long COVID: a living systematic review. *BMJ Glob Health* **6** (2021). https://doi.org:10.1136/bmjgh-2021-005427

7 Lopez-Leon, S. *et al.* More than 50 long-term effects of COVID-19: a systematic review and meta-analysis. *Sci Rep* **11**, 16144 (2021). https://doi.org:10.1038/s41598-021-95565-8
